# Power of PgR expression as a prognostic factor for ER-positive/HER2-negative breast cancer patients at intermediate risk classified by the Ki67 labeling index

**DOI:** 10.1186/s12885-017-3331-4

**Published:** 2017-05-22

**Authors:** Sasagu Kurozumi, Hiroshi Matsumoto, Yuji Hayashi, Katsunori Tozuka, Kenichi Inoue, Jun Horiguchi, Izumi Takeyoshi, Tetsunari Oyama, Masafumi Kurosumi

**Affiliations:** 1 Division of Breast Surgery, Saitama Cancer Center, Saitama, Japan; 2 Division of Breast Oncology, Saitama Cancer Center, Saitama, Japan; 3 Department of Thoracic and Visceral Organ Surgery, Gunma University Graduate School of Medicine, Gunma, Japan; 4 Department of Diagnostic Pathology, Gunma University Graduate School of Medicine, Gunma, Japan; 5 Department of Pathology, Saitama Cancer Center, 780 Komuro, Ina-machi, Kitaadachi-gun, Saitama, 362-0806 Japan

**Keywords:** ER-positive and HER2-negative breast cancer, Ki67 labeling index, Progesterone receptor, Prognosis

## Abstract

**Background:**

The Ki67 labeling index (LI) is regarded as a significant prognostic marker in ER-positive/HER2-negative breast cancer patients. The expression of PgR has recently been identified as another prognostic marker. In the present study, we investigated the prognostic utilities and most suitable cut-off values for Ki67 and PgR, and evaluated the relationship between Ki67 LI and PgR expression in ER-positive/HER2-negative breast cancer.

**Patients and methods:**

In the present study, 177 consecutive Japanese women with ER-positive/HER2-negative invasive carcinoma of no special type who were treated between 2000 and 2001 were enrolled. Recurrence-free survival (RFS) and cancer-specific survival (CSS) were analyzed according to Ki67 LI and PgR expression, and significant cut-off values for selecting patients with a poor prognosis were evaluated.

**Results:**

The cut-off values for Ki67 LI as a prognostic marker plotted against *P* values showed bimodal peaks at 10% and 30%. Among the cut-off points examined for the PgR status, 20% PgR positivity was the most significant for predicting survival differences (RFS: *P* = 0.0003; CSS: *P* < 0.0001). A multivariate analysis showed that PgR (≥20%) was an independent prognostic marker (RFS: *P* = 0.0092; CSS: *P* = 0.00014). Furthermore, in the intermediate risk group with Ki67 LI of 10–30%, the low PgR <20% group had a markedly poorer prognosis for RFS and CSS (RFS: *P* < 0.0001; CSS: *P* < 0.0001).

**Conclusions:**

The expression of PgR is a potent prognostic indicator for evaluating the long-term prognosis of ER-positive/HER2-negative breast cancer, and the most suitable cut-off value was found to be 20%. Furthermore, the PgR status is a powerful method for selecting patients with a poor prognosis among ER-positive/HER2-negative patients at intermediate risk, as assessed using Ki67 LI.

**Electronic supplementary material:**

The online version of this article (doi:10.1186/s12885-017-3331-4) contains supplementary material, which is available to authorized users.

## Background

Breast cancer has clinical and biological heterogeneity, and research is ongoing to detect potent indicators associated with cell growth and differentiation, which are involved in tumor formation and the progression of breast cancer. Breast cancer has recently been classified into 6 intrinsic subtypes: luminal A, luminal B, human epidermal growth factor receptor type 2 (HER2)-enriched, basal-like, claudin-low, and normal-like, using semi-unsupervised gene expression array analyses [1–3]. In routine practice, intrinsic subtypes have been obtained using immunohistochemical evaluations of the estrogen receptor (ER), progesterone receptor (PgR), HER2, and Ki67 labeling index (LI), and the following practical classification of intrinsic subtypes was proposed at the St. Gallen consensus meeting of breast cancer: luminal A-like type (ER-positiveand/or PgR-positive, HER2-negative, low proliferation, and low tumor burden), luminal B-like type (ER-positiveand/or PgR-positive, HER2-negative, high proliferation, and high tumor burden), hormone receptor-positive and HER2-positive type, hormone receptor-negative and HER2-postive type, and triple-negative (TN) type (hormone receptor-negative and HER2-negative) [4, 5].

Ki67 has been associated with cell cycle activity and is expressed at various levels during the G1, S, G2, and M phases [6]. Ki67 expression was found to correlate well with the growth fraction in various human cancers including breast cancer [7]. In previous studies, Ki67 LI was valued as a prognostic factor associated with ER-positive/HER2-negative breast cancer outcomes. Ki67 LI is also regarded as a biomarker for therapeutic decisions for ER-positive/HER2-negative breast cancer [8, 9]. However; definite cut-off values for Ki67 have not yet been decided, and evidence to indicate that patients with low Ki67 LI among those with ER-positive/HER2-negative breast cancer are at a lower risk of breast cancer relapse is limited [10, 11]. Dowsett et al., on behalf of the International Ki67 in Breast Cancer Working Group of the Breast International Group and North American Breast Cancer Group, provided an overview of the state of the art of Ki67 evaluations and proposed a set of guidelines for the analysis and reporting of Ki67 [12, 13]. They also suggested that a standardized method and value set need to be established for the evaluation of Ki67 [14]. Manual counting appears to be accepted, but represents a huge task for pathologists and is not highly reproducible. Hida et al. modified their method of a visual assessment to create a new 5-grade scale for the evaluation of Ki67 and verified its utility [15]. On the other hand, Perou et al. initially proposed a molecular classification for breast cancer [1, 2], and the subsequent expansion of this work into a larger cohort of patients showed that luminal B tumors had a poorer prognosis than Luminal A tumors despite treatments with hormonal therapy [16]. These discrepancies between luminal A and luminal B may be due to the different estrogen-related intracellular signaling pathways in breast cancer cells. However, many questions regarding distinguishing between the mechanisms responsible for luminal A and luminal B breast cancer, which lead to the proliferation and metastasis of breast cancer cells, remain unanswered [17]. Prat et al. reported that an empiric cut-off of more than 20% of PgR-positive tumor cells was statistically proven to be significant for predicting survival differences within luminal-type breast cancer defined by their molecular classification. They concluded that the new definition of the luminal A-like type was ER-positive/HER2-negative/Ki67 LI less than 14%/PgR more than 20% [18]. Therefore, PgR may be a useful indicator for classifying ER-positive/HER2-negative breast cancer between the luminal A-like subtype and B-like subtype [19].

However, the relationship between Ki67 LI and the expression of PgR has not yet been examined, and the utility of a combined evaluation method using these 2 factors for the selection of a poor prognosis group from ER-positive/HER2-negative breast cancer patients has not yet been established. In the present study, we investigated the prognostic utilities and most suitable cut-off values for Ki67 LI and PgR expression, and then analyzed the relationship between Ki67 LI and PgR expression as a prognostic marker in patients with ER-positive/HER2-negative breast cancer.

## Methods

### Patient backgrounds and eligibility

The paraffin-embedded samples of tumors from 272 consecutive patients with invasive breast cancer of no special type that were larger than 5 mm and diagnosed at Saitama Cancer Center between January 2000 and December 2001 were initially retrieved, the status of ER, PgR, HER2, and Ki67 LI were assessed, and the intrinsic subtypes of these patients were decided.

After the evaluation of intrinsic subtypes, 177 patients with ER-positive/HER2-negative breast cancer were selected and enrolled in this study. All patients underwent breast-conserving surgery or modified radical mastectomy without neoadjuvant chemotherapy or neoadjuvant endocrine therapy. We excluded patients with bilateral breast cancer and male breast cancer. The medical records of these ER-positive/HER2-negative patients were reviewed for clinicopathological characteristics including the pathological T and N status and American Joint Committee on Cancer (AJCC) stage, and follow-up data for all patients were obtained with a median follow-up period of 130 months (4–149 months).

This study was conducted in accordance with the Declaration of Helsinki, and the protocol of the study was approved by the Institutional Review Board of the Saitama Cancer Center. All patients enrolled in this study agreed to the scientific examination of tumor tissues obtained by surgery and provided written comprehensive informed consent.

### Procedures to examine ER, PgR, HER2, and Ki67

Buffered formalin-fixed paraffin-embedded specimens were cut into 4-μm-thick sections to be prepared for immunohistochemistry for ER, PgR, HER2, and Ki67 as well as dual HER2 in situ hybridization (DISH). The sources of primary antibodies were as follows: ER (1D5, DAKO, Denmark), PgR (PgR636, DAKO, Denmark), HER2 (HercepTest, DAKO, Denmark), and Ki67 (MIB-1, DAKO, Denmark). Immunohistochemistry for ER, PgR, and HER2 was performed manually using the streptavidin-biotin method. In patients with a HER2 score 2+ by immunohistochemistry, amplification of the HER2 gene was evaluated using the dual in situ hybridization (DISH) method with an automated slide processing system (BenchMark® XT, Ventana Medical Systems, Inc., Tucson, Arizona). Furthermore, immunohistochemistry for Ki67 was performed automatically using an automated immunohistochemistry instrument (BenchMark® XT, Ventana Medical Systems, Inc., Tucson, Arizona).

### Evaluation of ER, PgR, and HER2 status and Ki67 LI

**Fig. 1:**
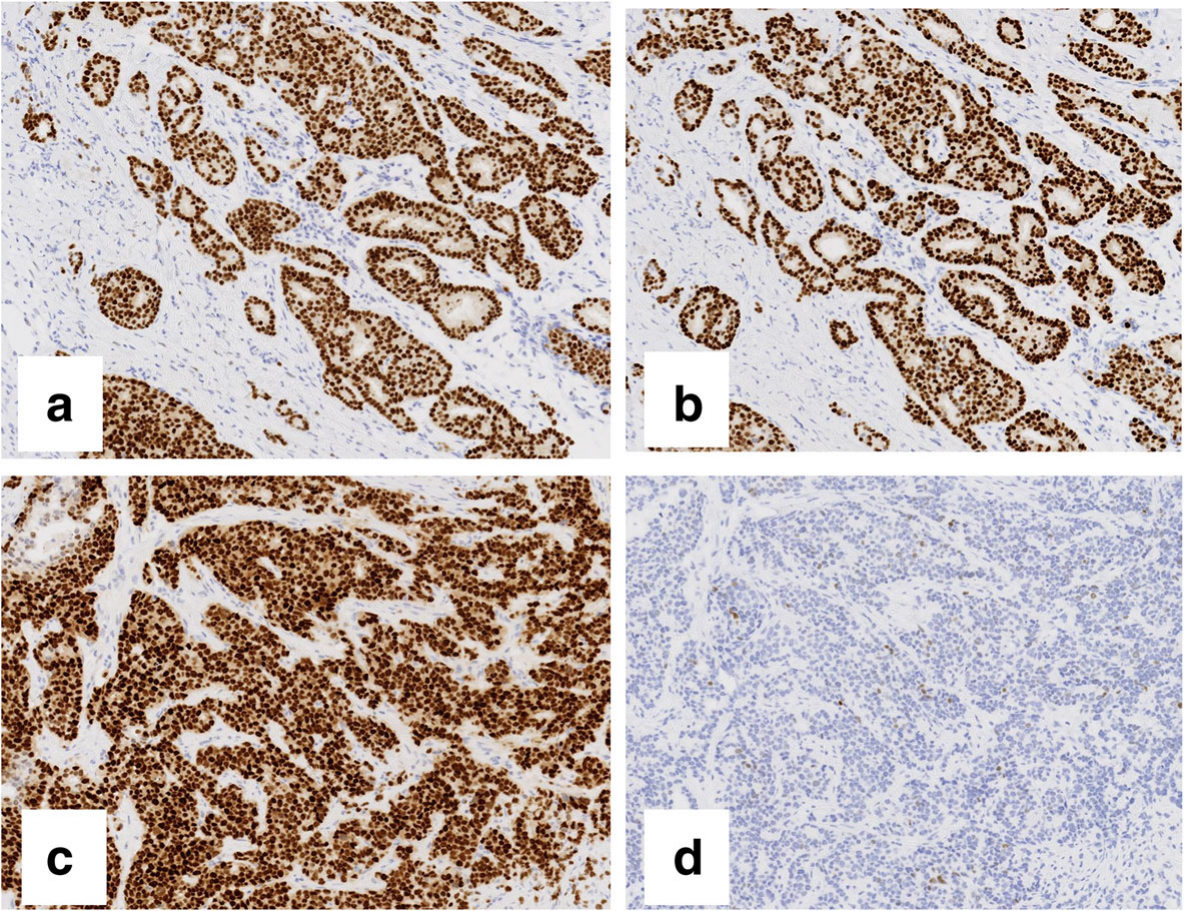
Combination patterns of ER and PgR expression. Case 1: ER-positive (≥1%) and high PgR expression breast cancer (**a** ER expression, **b** PgR expression) Case 2: ER-positive (≥1%) and low PgR expression breast cancer (**c** ER expression, **d** PgR expression)

The percentages of nuclei stained for ER and PgR were calculated (Fig. 1), and a patient was considered to be “positive” if the breast tumor contained at least 1% positive cells, in accordance with the American Society of Clinical Oncology (ASCO) and College of American Pathologists (CAP) criteria. In addition, the degrees of staining for ER and PgR were evaluated using the Allred score. In the Allred scoring system, proportion scores were defined as: 0 (0% staining), 1 (<1%), 2 (1-10%), 3 (10-33%), 4 33-67%), and 5 (>67%), while intensity scores were defined as: 0 (no staining), 1 (weak staining), 2 (intermediate staining), and 3 (strong staining). The total score was obtained by adding the proportion score and intensity score in order to attain final scores of 0 and 2–8. We also added a “20%” cut-off point to evaluate PgR staining. Since it has been reported that tumors with an Allred score ≤ 2 are hormone non-responsive [20], we evaluated breast cancer patients with an Allred score ≥ 3.

An evaluation of the HER2 status using immunohistochemistry and DISH was performed using the guidelines of ASCO/CAP proposed in 2013. Membranous staining for HER2 was graded as follows: scores 0, 1+, 2+, and 3+. Tumors with a score 2+ were subjected to an in situ hybridization (ISH) assay in order to assess the gene amplification of HER2. A HER2 score of 3+ or 2+ /DISH positive was defined as HER2-positive cancer. We excluded HER2-positive/ER-positive patients from further examination because their prognosis is worse and the strategy of treatment using HER2-targeting agents markedly differs from that of HER2-negative/ER-positive patients.

Images of Ki67 staining were captured using a digital pathology system (NanoZoomer 2.0-HT, C9600–13, Hamamatsu Photonics, Co., Japan) with viewer software (NDP.view2, Hamamatsu Photonics, Co., Japan), and photographs of the selected area were printed. Evaluations of Ki67 LI (percentage of positivity) were performed using printed photographs. We initially selected the representative area from the whole area of Ki67-stained sections. We principally observed the front line of the invasive region, and selected warm to hot spots in density for Ki67 labeling. The numbers of positive and negative nuclei stained by Ki67 immunohistochemistry were counted. At least 500 tumor cells were counted and Ki67 LI was calculated.

### Statistical analysis

Statistical analyses were conducted using SPSS v22.0 (IBM Corp., USA). The relationship between Ki67 LI and PgR expression (Allred score) was analyzed by Spearman’s rank correlation test. The Kaplan-Meier method and Log-rank test were used to estimate recurrence-free survival (RFS) and cancer-specific survival (CSS). RFS was defined as the length of time from the period of surgery to any recurrence (including ipsilateral breast recurrence). CSS was defined as the time from the day of surgery until the time of death due to the progression of breast cancer. RFS and CSS were compared between patients divided into two groups according to the degree of PgR staining and Ki67 LI. Significant cut-off values were obtained for the selection of patients with the worst prognosis based on the lowest *P* value derived from the survival analysis. In addition, some clinicopathological factors such as the menopausal status, pathological T status, pathological node status, histological grade, and type of adjuvant therapy were included in the multivariate survival analysis using a Cox proportional hazards regression model, and 95% confidence intervals were assessed for each factor. A *P* value < 0.05 was defined as being significant.

## Results

### Patient and tumor characteristics

Patient and tumor characteristics were shown in Table 1. The median age of the 177 patients enrolled in this study was 54 years (age range, 26–87 years); 162 patients (91.5%) were older than 41 years and 100 patients (56.5%) were post-menopausal. Seventy patients (39.5%) received adjuvant chemotherapy, while 146 (82.5%) received adjuvant endocrine therapy. The distribution of patients stratified by Allred scores and the proportion of PgR was shown in Table 2. The median Ki67 LI of all patients was 18.2% (index range, 0.8–74%), and the distribution of patients stratified by the Ki67 LI was also shown in Table 2. Forty-six patients (26.0%) were in the low Ki67 (less than 10%) LI group, while 33 (18.6%) were in the high Ki67 (more than 30%) LI group.

**Table 1:** Patient and tumor characteristics at baseline

	No. of patients	Percent
Total	177	100
Menopausal status at diagnosis		
Premenopausal	77	43.5
Postmenopausal	100	56.5
Pathological tumor size		
T 1	99	55.9
T 2	58	32.8
T 3	10	5.6
T 4	10	5.6
Pathological nodal status		
N 0	95	53.7
N 1	46	26.0
N 2	23	13.0
N 3	9	5.1
Not evaluated	4	2.3
Pathological stage		
I	63	35.6
II A	55	31.1
II B	22	12.4
III A	14	7.9
III B	10	5.6
III C	9	5.1
Not evaluated	4	2.3
Type of surgery		
Breast-conserving surgery	147	83.1
Mastectomy	30	16.9
Axillary management		
Sentinel lymph node biopsy alone	95	53.7
Axillary lymph node dissection	79	44.6
No surgery	3	1.7
Histological grade		
1	41	23.2
2	67	37.9
3	69	39.0
Adjuvant Chemotherapy		
Yes	70	39.5
No	107	60.5
Adjuvant Endocrine therapy		
Yes	146	82.5
No	31	17.5

**Table 2:** Distribution of PgR expression and the Ki67 labeling Index

	No	Percent
Allred Scores of PgR		
0	21	11.8
2	0	0.0
3	4	2.3
4	12	6.8
5	17	9.6
6	28	15.8
7	42	23.7
8	53	29.9
Proportion of PgR (%)		
0	21	11.8
> 0 and <1	4	2.3
≥ 1 and <10	17	9.6
≥ 10 and <20	23	13.0
≥ 20 and <33	10	5.6
≥ 33 and ≤67	48	27.1
> 67	54	30.5
Ki67 labeling index (%)		
≤ 10	46	26.0
> 10 and <14	20	11.3
≥ 14 and <20	33	18.6
≥ 20 an <30	45	25.4
≥ 30	33	18.6

### Survival analysis according to the status of PgR

**Fig. 2:**
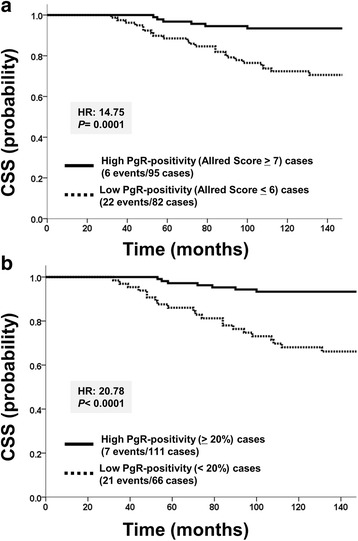
Survival curves stratified by PgR expression. **a** Comparisons of cancer-specific survival (CSS) between the high PgR positivity (Allred score ≥ 7) and low PgR positivity (Allred score ≤ 6) groups. **b** Comparisons of CSS between the high PgR positivity (≥20%) and low PgR positivity (<20%) groups

The hazard ratios of RFS and CSS stratified by the PgR status were evaluated using the Kaplan-Meier method and Log-rank test. The cut-off values for the PgR status and associated *P* values for the difference in the probability of survival between low and high PgR expression groups stratified by the Allred score were as follows: 0 vs 2–8, cut-off point 2 (RFS: HR = 5.88, *P* = 0.015; CSS: HR = 3.73, *P* = 0.053), 0–2 vs 3–8, cut-off point 3 (RFS: HR = 5.88, *P* = 0.015; CSS: HR = 3.73, *P* = 0.053), 0–3 vs 4–8, cut-off point 4 (RFS: HR = 5.43, *P* = 0.020; CSS: HR = 4.39, *P* = 0.036), 0–4 vs 5–8, cut-off point 5 (RFS: HR = 2.95, *P* = 0.086; CSS: HR = 2.72, *P* = 0.099), 0–5 vs 6–8, cut-off point 6 (RFS: HR = 3.59, *P* = 0.058; CSS: HR = 4.35, *P* = 0.037), 0–6 vs 7–8, cut-off point 7 (RFS: HR = 8.68, *P* = 0.0032; CSS: HR = 14.75, *P* = 0.0001), and 0–7 vs 8, cut-off point 8 (RFS: HR = 5.68, *P* = 0.017; CSS: HR = 4.06, *P* = 0.044). The most significant cut-off point for prognosis was between the group with a score 0–6 and the group with a score 7–8, cut-off point 7 (Fig. 2a).

In addition, the cut-off points for the PgR status and associated *P* values for the difference in the probability of survival between the low and high PgR expression groups stratified by the percentage of positive cells (%) were as follows: 0% (RFS: HR = 5.88, *P* = 0.015; CSS: HR = 3.73, *P* = 0.053), 1% (RFS: HR = 7.08, *P* = 0.00078; CSS: HR = 6.47, *P* = 0.011), 10% (RFS: HR = 5.45, *P* = 0.020; CSS: HR = 4.51, *P* = 0.034), 20% (RFS: HR = 13.33, *P* = 0.0003; CSS: HR = 20.78, *P* = 0.000005), 33% (RFS: HR = 9.98, *P* = 0.0016; CSS: HR = 14.98, *P* = 0.0001), and 67% (RFS: HR = 6.7, *P* = 0.014; CSS: HR = 4.31, *P* = 0.038). The most significant cut-off point for prognosis was 20% (Fig. 2b).

### Survival analysis according to Ki67 LI

**Fig. 3:**
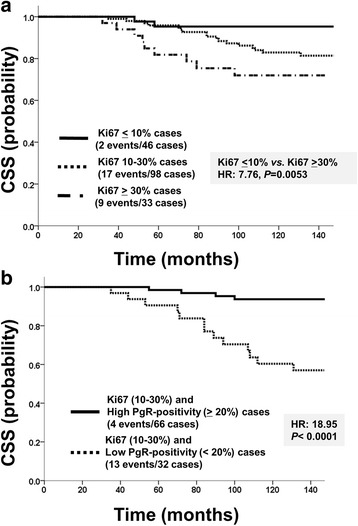
Survival curves stratified by the combination tool using the expression of PgR and Ki67. **a** Relationship between the Ki67 labeling index and cancer-specific survival (CSS). **b** Survival curves stratified by PgR expression according to staining percentages in the intermediate Ki67 labeling index (Ki67 > 10 and <30%) group. Comparisons of CSS between the PgR-positive (≥20%) and PgR-negative (<20%) groups

The hazard ratios of RFS and CSS stratified by Ki67 LI were assessed using the Kaplan-Meier method and Log-rank test. The cut-off values for Ki67 LI and associated *P* values for the difference in the probability of survival between the high Ki67 and low Ki67 groups were as follows: 10% (RFS: HR = 2.77, *P* = 0.096; CSS: HR = 5.21, *P* = 0.022), 14% (RFS: HR = 3.57, *P* = 0.059; CSS: HR = 4.77, *P* = 0.029), 18% (RFS: HR = 2.13, *P* = 0.14; CSS: HR = 1.98, *P* = 0.16), 20% (RFS: HR = 1.67, *P* = 0.20; CSS: HR = 3.46, *P* = 0.063), and 30% (RFS: HR = 2.66, *P* = 0.010; CSS: HR = 4.63, *P* = 0.031). Cut-off values for Ki67 LI as a prognostic marker plotted against *P* values showed bimodal peaks at 10% and 30%. These results allowed patients to be classified into 3 groups using the cut-off values of Ki67 as follows: a) low Ki67 LI group, Ki67 LI: ≤10%; b) intermediate Ki67 LI group, Ki67 LI: >10 and <30%; and c) high Ki67 LI group, Ki67 LI: ≥30%. The survival rates of the 3 groups were significantly different in CSS, but not in RFS (RFS: HR = 4.28, *P* = 0.12; CSS: HR = 7.77, *P* = 0.021; Fig. 3a).

### Relationship between the expression of PgR and Ki67 LI

No correlation was observed between Ki67 LI and PgR expression (*P* = 0.814). The survival of the high Ki67 LI group was significantly worse than that of the low Ki67 LI group (RFS: HR = 4.04, *P* = 0.044; CSS: HR = 7.76, *P =* 0.0053; Fig. 3a). However, it was difficult to determine the prognosis of the intermediate Ki67 LI group, in which as many as 98 (55.4%) ER-positive/HER2-negative breast cancer patients were classified. In the intermediate Ki67 LI group, the low PgR group had a markedly poorer prognosis for RFS and CSS (RFS: HR = 16.60, *P* = 0.000046; CSS: HR = 18.95, *P =* 0.000013; Fig. 3b). The intermediate group was clearly divided according to Ki67 with the addition of PgR into two distinctive prognostic subgroups.

### Relationships between prognosis and clinicopathological characteristics of tumors

A univariate analysis identified the negative expression of PgR, high Ki67 LI, high histological grade (grade 1/2 vs. 3; RFS: HR = 3.69, *P* = 0.055; CSS: HR = 6.44, *P* = 0.011), high pathological T stage (pathological T 1/2 vs. pathological T 3/4; RFS: HR = 10.74, *P* = 0.0011; CSS: HR = 8.90, *P* = 0.0029), and positive pathological node status (negative vs. positive, RFS: HR = 16.94, *P* = 0.000039; CSS: HR = 10.72, *P* = 0.0011) as worse prognostic factors in this study. The menopausal status or receiving adjuvant endocrine therapy, which we consider as important factors to treat ER-positive/HER2-negative breast cancer, did not correlate with prognosis in this study. Receiving adjuvant chemotherapy correlated with prognosis in this study (adjuvant chemotherapy no vs. yes; RFS: HR = 5.07, *P* = 0.024; CSS: HR = 3.67, *P* = 0.055), however; a multivariate analysis confirmed that receiving adjuvant chemotherapy did not correlate with prognosis (adjuvant chemotherapy no vs. yes; RFS: HR = 13.7, *P* = 0.35; CSS: HR = 1.25, *P* = 0.59). On the other hand, a multivariate analysis (Table 3) showed that PgR (cut-off value: 20%) was an independent prognostic marker for RFS and CSS (RFS: HR = 2.33, *P* = 0.013; CSS: HR = 5.15, *P* = 0.00045). Based on the results of the multivariate analysis, the pathological lymph node status was also identified as an independent prognostic marker for RFS and CSS (RFS: HR = 3.16, *P* = 0.0022, CSS: HR = 2.69, *P* = 0.042).

**Table 3:** Results of a multivariate survival analysis using a Cox proportional hazards regression mode on the influence of clinicopathological variables including PgR and Ki67

	RFS			CSS		
Characteristics	HR	95% CI	*P*	HR	95% CI	*P*
PgR expression						
≥ 20%	Referent					
< 20%	2.33	1.19–4.54	0.013	5.15	2.06–12.85	0.00045
Ki67 labeling index						
≤ 10%	Referent					
> 10 and <30%	0.52	0.18–1.53	0.24	0.28	0.05–1.44	0.13
≥ 30%	0.69	0.33–1.47	0.34	0.68	0.29–1.57	0.36
Menopausal status						
Pre-	Referent					
Post-	1.24	0.63–2.43	0.53	0.72	0.33–1.61	0.43
Pathological T stage						
T 1–2	Referent					
T 3–4	1.49	0.68–3.25	0.32	1.51	0.58–3.90	0.40
Pathological N stage						
N 0	Referent					
N 1–3	3.16	1.51–6.58	0.0022	2.69	1.04–6.99	0.042
Histological grade						
1,2	Referent					
3	1.31	0.67–2.56	0.43	1.40	0.60–3.25	0.44
Adjuvant chemotherapy						
No	Referent					
Yes	1.37	0.71–2.64	0.35	1.25	0.55–2.82	0.59
Adjuvant endocrine therapy						
No	Referent					
Yes	1.15	0.39–3.37	0.80	1.20	0.27–5.42	0.81

Abbreviations: *RFS* recurrence-free survival, *CSS* cancer-specific survival, *HR* hazard ratio, *95% Cl* 95% Confidence interval, *PgR* progesterone receptor

In addition, a multivariate analysis on the intermediate Ki67 LI group showed that PgR (cut-off value: 20%) was an independent potent prognostic marker for RFS and CSS (RFS: HR = 4.67, *P* = 0.00052; CSS: HR = 11.66, *P* = 0.00026) (Additional file 1).

## Discussion

It has been known that the positive rate of ER and/or PgR in breast cancer is approximately 70% [21], and ER is considered to have key functions in the development and progression of breast cancer. In addition, ER regulates many gene and protein actions within genomic and non-genomic pathways. Furthermore, estrogen signals mediated by ER control the genomic pathway which works as a transcription factor for targeted genes, and ER is activated by the signal crosstalk between estrogen and growth factors such as epidermal growth factor and insulin growth factor-1 via transmembrane receptor phosphorylation [22, 23]. On the other hand, PgR induced by ER acts as a key factor in induction, progression and maintenance of the neoplastic phenotype of ER-positive breast cancer [24]. Recent clinical findings demonstrated that the PgR status needs to be considered when discussing relative-risk reductions expected from endocrine treatments in individual patients [25]. In the present study, we revealed that the extent of PgR expression was a potent prognostic indicator for evaluating the long-term prognosis of ER-positive/HER2-negative breast cancer and that the most suitable cut-off value was 20%, which was consistent with previous findings [18]. Further research is needed in order to elucidate the biological mechanisms underlying the relationship between PgR expression and the prognosis of ER-positive/HER2-negative breast cancer patients.

We also classified ER-positive/HER2-negative breast cancer more simply into the following 3 types according to the percentages of Ki67 LI: Ki67 ≤ 10%; Ki67 > 10 and <30%; and Ki67 ≥ 30%. This Ki67 classification correlated with the long-term survival of patients with ER-positive/HER2-negative breast cancer. On the basis of these results, we classified ER-positive/HER2-negative breast cancer patients into 3 risk groups: low, intermediate, and high risk. In addition, we selected adjuvant therapeutic options for low and high risk groups, such as hormone therapy alone for low risk patients and chemo-endocrine therapy for high risk patients. However, difficulties have been associated with establishing a strategy for adjuvant therapy for the intermediate risk group, which accounts for more than 50% of ER-positive/HER2-negative breast cancer patients.

In the St. Gallen consensus meeting of 2015, they showed that hormone receptor-positive/HER2-negative breast cancer may be divided into the luminal A-like type (high ER/PgR and clearly low Ki67), luminal-B like type (low ER/PgR and clearly high Ki67), and intermediate type. They suggested that Ki67 scores needed to be interpreted based on local laboratory values; if a laboratory has a median Ki67 LI of 20%, values of 30% or more may be regarded as high, while those of 10% or less are clearly low [5]. We also confirmed that the survival of the Ki67 LI high (≥30%) group was significantly worse than that of the Ki67 LI low (≤10%) group. On the other hand, the intermediate type was defined as an uncertain type regarding the degree of risk and responsiveness to endocrine therapy and chemotherapy. They suggested that in the intermediate risk type of ER-positive/HER2-negative breast cancer, multi-parameter molecular tests may be used if available. Genomic and clinical variables both need to be included in a common algorithm in order to yield the most accurate prediction model in ER-positive/HER2-negative breast cancer [26]. The results of the present study indicate that the low PgR (<20%) group has a markedly poorer prognosis among patients with ER-positive/HER2-negative and intermediate Ki67 LI breast cancer. Maisonneuve et al. also suggested that patients with tumors with the intermediate type (Ki67 LI: 14% to 19%) and low PgR (<20%) expression had similar outcomes to those of patients with luminal B-like breast cancer [27]. This combination tool using PgR and Ki67 LI may be valuable for selecting patients with a good prognosis in intermediate type ER-positive/HER2-negative breast cancer.

For decision of appropriate cut-off values for PgR, it might be necessary to obtain data from large-scale validation studies, but a few studies have been published on the PgR status. Prat et al. recently reported that an empirical cut-off of more than 20% for PgR-positive tumor cells was statistically proven to be significant for predicting survival differences among 2257 luminal-type breast cancer patients defined by their molecular classification [18]. Furthermore, Mohammed et al. revealed that PgR gene loss was an independent potent prognostic marker for survival using TCGA data [28]. However, the novel results obtained in the present study may be limited by the PgR cut-off values selected, and, thus, further prospective and large-scale clinical research appears to be necessary in order to confirm the most suitable cut-off value for PgR expression as a prognostic factor for the Ki67-intermediate group in ER-positive/HER2-negative breast cancer patients.

## Conclusions

The extent of PgR expression as well as Ki67 LI may be a potent prognostic indicator for evaluating the long-term prognosis of ER-positive/HER2-negative breast cancer. The results of the present study suggest that examining the extent of PgR expression allows for the selection of patients with a poor prognosis and that the most suitable cut-off value was 20%. Furthermore, PgR expression and Ki67 LI represent a powerful method for selecting patients with a poor prognosis among those with ER-positive/HER2-negative breast cancer.

## Additional file

Additional file 1: Results of a multivariate survival analysis on the influence of clinicopathological variables including PgR in the intermediate Ki67 labeling index group. (PDF 118 kb)

## Abbreviations

AJCC: American Joint Committee on Cancer Staging System
ASCO: American Society of Clinical Oncology
CAP: College of American Pathologists
ER: Estrogen receptor
HER2: Human epidermal growth factor receptor 2;
HR: Hazard ratio.
Ki67 LI: Ki67 labeling index
OS: Overall survival
PgR: Progesterone receptor
RFS: Relapse-free survival
