# Supplementary material for: Power of PgR expression as a prognostic factor for ER-positive/HER2-negative breast cancer patients at intermediate risk classified by the Ki67 labeling index
Source: BMC Cancer. 2017 May 22;17:354. doi: 10.1186/s12885-017-3331-4 (PMC5441075; doi:10.1186/s12885-017-3331-4)
Supplement: Additional file 1: — Results of a multivariate survival analysis on the influence of clinicopathological variables including PgR in the intermediate Ki67 labeling index group. (PDF 118 kb) [file 12885_2017_3331_MOESM1_ESM.pdf]

Additional File 1

Results of a multivariate survival analysis on the influence of clinicopathological variables including PgR in the intermediate Ki67 labeling index group

	RFS			CSS		
Characteristics	HR	*P*	95%CI	HR	*P*	95%CI
PgR expression						
$\geq$ 20%	Referent					
< 20%	4.72	0.00042	1.99-11.19	11.09	0.00025	3.06-40.18
Menopausal status						
Pre-	Referent					
Post-	1.18	0.71	0.50-2.79	0.41	0.15	0.12-1.39
Pathological T stage						
T 1-2	Referent					
T 3-4	1.19	0.75	0.41-3.45	0.31	0.18	0.05-1.75
Pathological N stage						
N 0	Referent					
N 1-3	3.17	0.020	1.20-8.39	2.64	0.10	0.83-8.41
Histological grade						
1,2	Referent					
3	1.25	0.59	0.56-2.82	2.44	0.11	0.81-7.36
Adjuvant chemotherapy						
No	Referent					
Yes	0.81	0.63	0.35-1.90	0.65	0.40	0.24-1.77
Adjuvant endocrine therapy						
No	Referent					
Yes	0.56	0.39	0.15-2.11	1.41	0.75	0.17-11.91

Abbreviations:

RFS, Recurrence-free survival; CSS, Cancer-specific survival; HR, Hazard Ratio; 95% Cl, 95% Confidence interval; PgR, progesterone receptor.
